# CTF: a CRF-based transcription factor binding sites finding system

**DOI:** 10.1186/1471-2164-13-S8-S18

**Published:** 2012-12-17

**Authors:** Yupeng He, Yizhe Zhang, Guangyong Zheng, Chaochun Wei

**Affiliations:** 1 School of Life Sciences and Biotechnology, Shanghai Jiao Tong University, 800 Dongchuan Road, Shanghai 200240, China; 2 Key Laboratory of Systems Biology, Shanghai Institutes for Biological Sciences, Chinese Academy of Sciences, 320 Yueyang Road, Shanghai 200031, China; 3 Shanghai Center for Bioinformation Technology, 1278 Keyuan Road, Pudong District, Shanghai 201203, China; 4 Bioinformatics and Systems Biology Program, University of California, San Diego, 9500 Gilman Dr., La Jolla, CA 92093, USA

## Abstract

**Background:**

Identifying the location of transcription factor bindings is crucial to understand transcriptional regulation. Currently, Chromatin Immunoprecipitation followed with high-throughput Sequencing (ChIP-seq) is able to locate the transcription factor binding sites (TFBSs) accurately in high throughput and it has become the gold-standard method for TFBS finding experimentally. However, due to its high cost, it is impractical to apply the method in a very large scale. Considering the large number of transcription factors, numerous cell types and various conditions, computational methods are still very valuable to accurate TFBS identification.

**Results:**

**In this paper, we proposed a novel integrated TFBS prediction system, CTF, based on Conditional Random Fields (CRFs)**. Integrating information from different sources, CTF was able to capture patterns of TFBSs contained in different features (sequence, chromatin and etc) and predicted the TFBS locations with a high accuracy. We compared CTF with several existing tools as well as the PWM baseline method on a dataset generated by ChIP-seq experiments (TFBSs of 13 transcription factors in mouse genome). Results showed that CTF performed significantly better than existing methods tested.

**Conclusions:**

CTF is a powerful tool to predict TFBSs by integrating high throughput data and different features. It can be a useful complement to ChIP-seq and other experimental methods for TFBS identification and thus improve our ability to investigate functional elements in post-genomic era.

Availability: CTF is freely available to academic users at: http://cbb.sjtu.edu.cn/~ccwei/pub/software/CTF/CTF.php

## Introduction

Functional elements in genomes play important roles in many biology processes. For example, enhancers, silencers, and transcriptional factor binding sites (TFBSs) are required in transcription. Thus, identifying functional elements in genomes is one of most important problems in post-genomic era [1-3], which is essential to elucidate gene regulation comprehensively. TFBS is one important type of functional elements. However, it is very challenging to locate the actual positions of TFBSs because they are generally very short (10 ~ 20 bp) and highly degenerate. Besides, only a small fraction of their patterns in a genome are actually bounded by transcription factors [4-6].

Recently, the advance of experimental technology greatly expands our ability to detect the locations of TFBSs. ChIP-seq (chromatin immunoprecipitation followed by massively parallel sequencing) [7] technology is utilized to find out the binding motifs in a high accuracy and a high throughput. ChIP-seq is becoming the gold-standard method for TFBS identification. However, it has several limitations: 1). the quality and source of the antibody have a big impact on the result and it is hard to obtain high quality antibodies for all TFs; 2). its resolution (about 300 bps) is too low [8] to locate TFBSs, which are only about 20 bps; 3). Another major limitation is that ChIP-seq could detect the binding sites of only one transcription factor in one experiment and it is expensive. Although recent study showed that it was possible to identify binding sites of more than one TFs using a single ChIP-seq experiment [9], the cost is still prohibitively expensive to identify binding sites of many TFs in various cell types and conditions. Thus, computational methods are required as complementary means for TFBS identifying.

Efforts have been made to predict TFBSs computationally by searching patterns of TFBSs in genome. Position weight matrix (PWM) [10], which contains TFBS patterns in sequence level, is the most widely used model to represent and identify TFBSs. However, since the motifs are very short and typically degenerated, PWM alone is not discriminative enough and will predict a large number of false positives. Recently, various approaches have been proposed to reduce false positives by integrating information from other sources [11-14]. For example, histone markers were shown to correlate with transcription factor binding sites and were able to improve the accuracy significantly [13,15]. However, the co-occurrence of histone markers was not considered in all these methods mentioned above. The co-occurrence of histone markers was shown to reflect the state of chromatin and correlated with the binding events of transcription factors[16].

**Figure 1:**
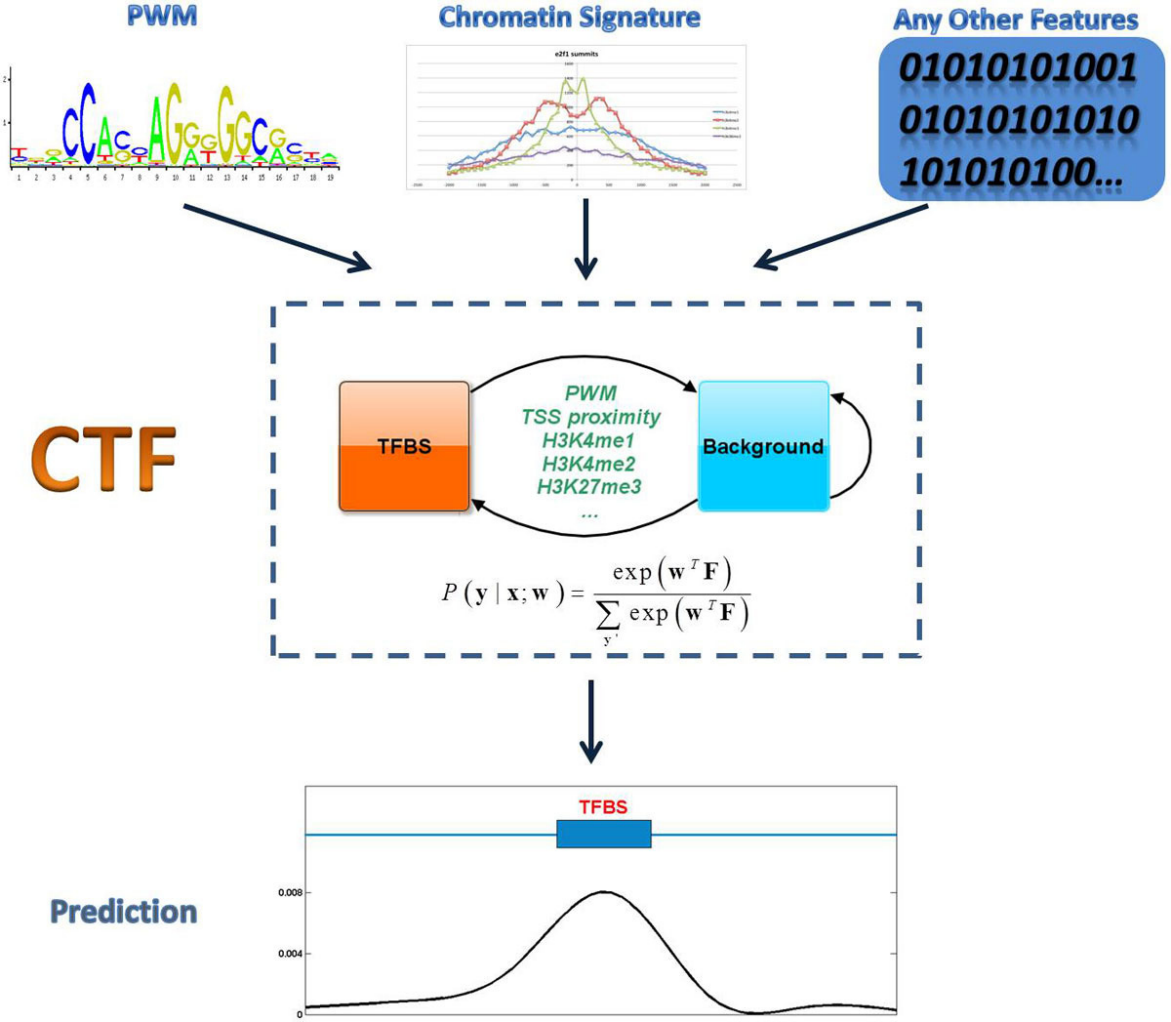
**System diagram of CTF**. This figure showed the system diagram of CTF. Different features (PWM, chromatin signatures, and any other features such as the distance to a TSS) were integrated into CTF. Combining these features, CTF called CRF framework to obtain the probability-like scores of TFBSs across the whole genome. The CRF model of CTF contained two states: transcription factor binding site (TFBS) state and non-TFBS (background) state.

In this paper, we present CTF (CRF-based TFBS Finding system), a novel method to identify TFBSs. Figure 1 showed the system diagram of CTF. Conditional Random Field (CRF) framework [17,18] was employed as the underlying model of CTF. CRF was introduced to bioinformatics area recently, such as gene prediction[19,20], and present promising results. CRF can capture sophisticated dependency and integrate information from different sources. Therefore it is an ideal framework for TFBS prediction.

Three types of features, the Position Weight Matrix (PWM), the distance to Transcription start sites (TSS proximity), and histone markers (8 distinct histone modifications), have been integrated into CTF (See Additional file 1 for more details). Test datasets were collected for13 transcription factors in mouse Embryonic Stem cells (ES cells). It is shown that by integrating PWM, histone markers and TSS proximity, CTF is able to predict TFBSs with high accuracy and it outperforms existing methods, including Chromia[13] and Cluster-Buster[21] significantly.

## Results

### Accurately predicting TFBSs by integrating PWM, TSS proximity and chromatin signature

**Figure 2:**
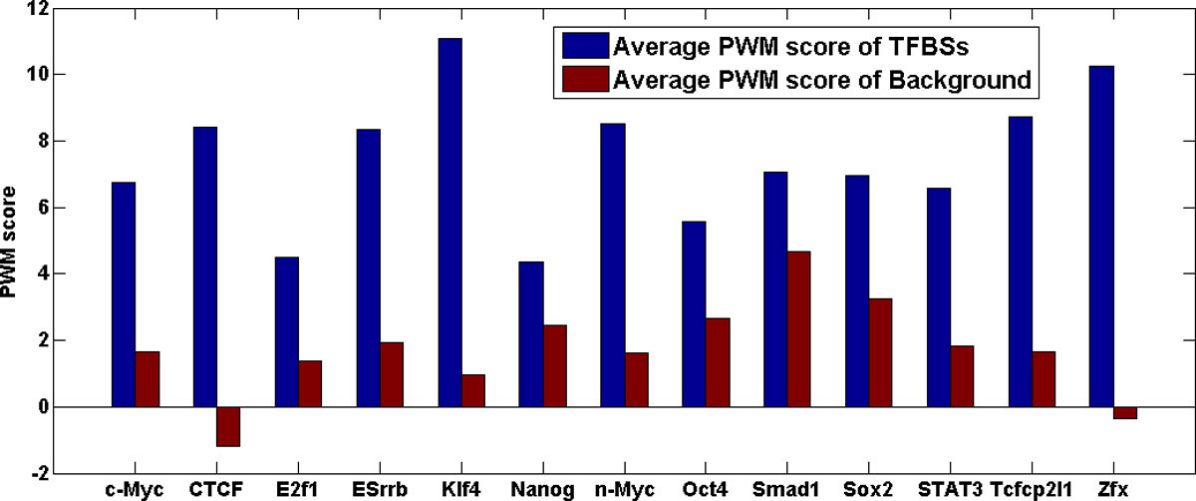
**PWM score comparison for TFBSs and background sequences**. This figure showed the average position weight matrix scores in bins that contain transcription factor binding sites (TFBSs) and bins that do not (background) on the datasets of 13 transcription factors.

CTF was evaluated on 13 TFBS datasets. Several features were assessed. First, traditional position weight matrix (PWM) model was used. Figure 2 shows the average PWM score in bins with or without TFBS inside. Those with TFBSs were with higher PWM scores, especially for the binding sites of CTCF, Klf4 and Zfx. Still, the PWM scores of binding sites of Smad1, Sox2 and Nanog failed to distinct themselves from the background.

CTF also integrated histone markers and transcription start site (TSS) data. Histone modifications were observed across genome and some of them correlated strongly with TFBSs[13]. In addition, by studying the combination of different histone modifications, it was shown that chromatin states were related to activity of genomic regions and regulation events[11]. Therefore, histone markers and their combinations were informative for the prediction of TFBSs. In our work, 8 distinct histone markers were used: H3K27me3, H3K36me3, H3K4me1, H3K4me2, H3K4me3, H3K9me3, H3 and H4K20me3. Another feature included was the TSS proximity. It was an indicator of whether a bin was within 2 kb of a TSS, the promoter region defined in this paper. The discriminative power of each histone marker could be measured by counting the frequency difference of a certain feature in bins with TFBSs and in bins without TFBSs. As Won et al presented that H3K4me2 and H3K4me3 were the most discriminative, while H3K4me1 was less discriminative [13]. It was consistent with our knowledge that H3K4me1/2/3 were active markers. In addition, we have observed the enrichment of binding sites of some TFs such as c-Myc and Zfx in promoter regions (Additional file 3) and the enrichment can be captured by the TSS-proximity feature.

**Figure 3:**
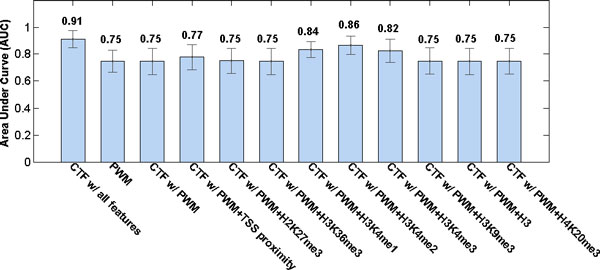
**Accuracy (AUC) for PWM and CTF with different features**. The figure showed the average AUCs (area under curve) of different models on the dataset of 13 TFBSs. Models included PWM and CTF with different combinations of features. The AUCs of CTF models were computed using 10-fold cross-validation, while the AUC of PWM was measured directly.

To evaluate the contribution of each feature, we tested CTF models that combined different features. In consistent with previous analysis, CTF with PWM and H3K4me1 (AUC = 0.84) or H3K4me2 (AUC = 0.86) or H3K4me3 (AUC = 0.82) showed superior performance than CTF with PWM and any other single feature (Figure 3). Also, integration of TSS proximity was able to improve the accuracy (AUC = 0.77) compared with model based solely on PWM (AUC = 0.75). Though, some other features made little contribution and related models showed similar performance as the baseline method that solely based on PWM. In the final combination, all features were included. We did not select features because the number of motifs in our dataset was very large which made it possible to include many features with a low risk of over fitting. Also, during the training of CTF, unrelated features would be assigned with weights close to zero. Combining all features, the final CTF (AUC = 0.91) outperformed all models with less features by at least 5%. This result demonstrated that CTF was able to integrate different information effectively and make better prediction.

### Comparison with other methods

To further evaluate CTF, we then compared CTF with a couple of prevalent existing algorithms. Chromia[13] is an integrated method based on Hidden Markov Model (HMM) and it predicts TFBSs based on PWM and chromatin signatures. Cluster-Buster[21] is a algorithm to find motif clusters (or cis-regulatory modules), which is also based on PWM. Cluster-Buster considers not only the signal (PWM score) of motifs but also their co-occurrence.

**Figure 4:**
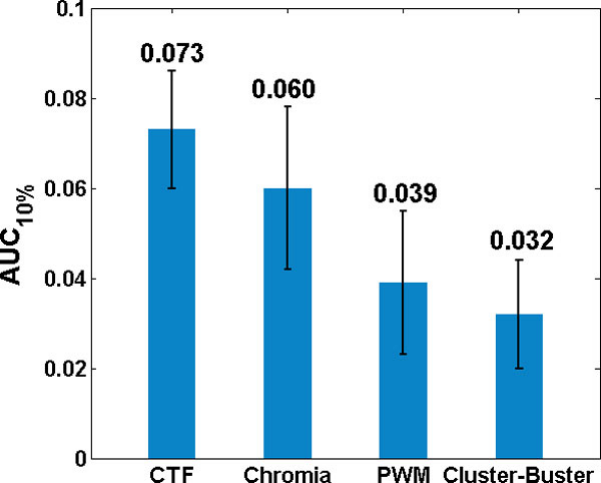
**Accuracy (AUC10%) for different TFBS identification methods**. This figure showed the average AUC_10% _(see Methods for details) of different TFBS prediction tools on the dataset of 13 TFBSs. The AUC of CTF models was computed using 10-fold cross-validation. The AUCs of other methods were measured directly from their results.

These tools were tested on the 13 TFBS datasets. The AUC_10% _was calculated as the measurement of performance (See Methods for details). Figure 4 present the accuracy distribution for different TFBS identification methods on the 13 datasets. Results showed that CTF achieved significantly better performance (AUC = 0.073) than all other methods. Also, the results showed CTF and Chromia outperformed PWM method, which implied that integration of histone markers was necessary and could indeed significantly improve the accuracy. Surprisingly, Cluster-Buster showed slightly worse AUC_10% _than PWM. However, the results of Cluster-Buster on Sox2 and Oct4 were slightly better than PWM. It was known that Sox2, Oct4 and Nanog were able to form a complex and their motifs were very close to each other.

**Figure 5:**
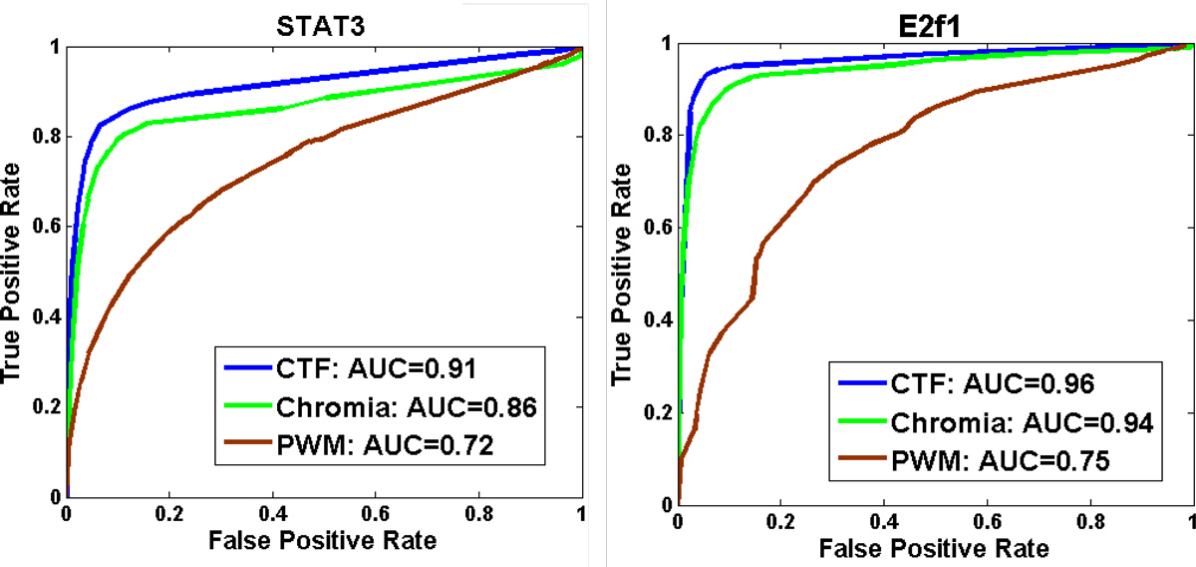
**ROC for CTF, Chromia and PWM on data of STAT3 and E2f1**. This figure showed the ROCs of CTF, Chromia and PWM on the dataset of E2f1 (left) and STAT3 (right). CTF was the CTF model with all features and its ROC was obtained by using 10-fold cross-validation and changing the threshold. ROC of Chromia was calculated using the data and model contained in its release. ROC of PWM was got by scoring directly. The complete list of results for all 13 TFs were shown in Additional file 2.

From previous results, it turned out that only Chromia was comparable to CTF in terms of AUC_10% _on test datasets. Then, we compared CTF, Chromia and PWM in terms of complete AUC as well as the true positive rate at 1% false positive rate. PWM was used as the baseline method. ROC curves of all three methods on data of STAT3 and E2f1 were shown in Figure 5 and results on all TFs were shown in Additional file 2. Results showed that CTF had better accuracy than other two methods. Table 1 listed the results of all 13 TFBS datasets. CTF performed the best in all datasets. On average, AUC of CTF was larger than AUC of Chromia by 3%. Next, we also compared the true positive rate (TPR) of all three methods at 1% false positive rate (FPR). The results were shown in Additional file 3. On average, the CTF had the highest TPR (0.55), which was much better than TPR of other two methods (TPR_Chromia _= 0.33 and TPR_PWM _= 0.23). To sum up, CTF outperformed existing methods in different metrics.

**Table 1:** The AUC of CTF, Chromia and PWM on the dataset of 13 TFs

	CTF	Chromia	PWM
c-Myc	**0.98**	0.94	0.84

CTCF	**0.76**	0.69	0.76

E2f1	**0.96**	0.94	0.75

Esrrb	**0.89**	0.84	0.77

Klf4	**0.96**	0.92	0.83

Nanog	**0.83**	0.82	0.62

n-Myc	**0.97**	0.94	0.86

Oct4	**0.92**	0.88	0.61

Smad1	**0.92**	0.89	0.66

Sox2	**0.90**	0.87	0.70

STAT3	**0.91**	0.86	0.72

Tcfcp2l1	**0.88**	0.83	0.79

Zfx	**0.97**	0.96	0.82

**Average**	**0.91**	0.88	0.75

## Discussion

CTF, a novel integrative TFBS prediction system, was proposed in this paper. Although CTF achieved a high accuracy, there are still much room for improvement. For example, in current version, only the locations of the peaks of histone modifications were considered in CTF. Continuous feature functions that score the shape and intensity could be included in the future versions. In addition, the CRF framework itself is very flexible and new features can be added into CTF in a straightforward manner. CTF can also be applied to similar problems such as the prediction of enhancers. We expect that CTF can facilitate the identification of binding sites of transcription factors as well as other functional elements, and improve our knowledge about gene regulation.

## Conclusions

In this paper, we present and evaluated CTF, a novel integrative method to predict transcription factor binding sties (TFBSs) by combining various features using conditional random field as the underlying framework. Our results showed that CTF successfully integrated position weight matrix (PWM), distance to transcription start sites (TSSs) and 8 distinct histone markers, which in total improved accuracy of TFBS prediction significantly. It outperformed models with only part of those features. Most importantly, when compared with some existing representative tools, CTF showed significant superior performance. CTF is an effective novel integrative TFBS prediction system, and has a great potential in other functional element finding.

## Methods

### CRF-based TFBS finding system

CTF system has been created to predict transcription factor binding sites (TFBSs) by integrating information from different sources. The system diagram of CTF was shown in Figure 1. In CTF, a genome is divided into 200 bp bins first. Then, the conditional probability-like score of a label sequence (TFBS and non-TFBS) given an observation sequence was computed as follows.

$$p\left(y|x;\lambda \right)=\frac{\text{exp}\left(\overset{L}{\underset{t=1}{\sum }}\overset{K}{\underset{k=1}{\sum }}\lambda_{k}f_{k}\left(y_{t},y_{t-1},t,x\right)\right)}{\underset{y^{\prime }}{\sum }\text{exp}\left(\overset{L}{\underset{t=1}{\sum }}\overset{K}{\underset{k=1}{\sum }}\lambda_{k}f_{k}\left(y_{t}^{\prime },y_{t-1}^{\prime },t,x\right)\right)}$$

where *y *is *the *label sequence or annotation of all bins, *x is the *observed genomic sequence *f_x _*is the k^-th ^feature functions and *λ_k _*is the corresponding weight. The feature function *f_k _*can be an arbitrary function on *x *and *y' *is any label sequence. In CTF, the possible values for label sequence of one bin is 0 (non-TFBS) and 1 (TFBS).

### Feature design

In CTF, several types of feature functions have been designed to capture patterns contained in features. The first type of feature functions are PWM scoring functions. The second type of feature functions are indicator functions. Each of these indictor function checks the occurrence of a feature. For example, a feature function of this type can be interpreted as an indicator of a bin in a promoter region if the *i*-th feature is TSS proximity, or an indicator of a bin within an H3K4me2 peak if that *i*-th feature corresponds to H3K4me2. The third type of feature functions targets the co-occurrence of two histone markers. This type of feature functions are able to capture co-occurring features such as a bivalent domain[22] or a bin that is "not in a promoter region or H3K4me3", which is a marker of active enhancer[23]. In addition, we have defined feature functions to capture patterns in adjacent bins as a complement for the above feature functions. With these feature functions, CTF is able to distinguish TFBSs from the background with high accuracy.

Different function templates were created for different types of feature functions in CTF. Let **x **be a feature matrix (note, x is not a genomic sequence here. See Additional file 1 for more details), then *x_i, j _*is the element in *i*-th row and *j*-th column, i.e. the value of *i*-th feature in the *j*-th bin in the genome. The first row corresponds to PWM scores. Similarly, **y **is the label sequence (or annotation sequence) and *y_j _*is the label of the *j*-th bin (1 for TFBS and 0 otherwise). *I*{*conditions*} is denoted as an indicator function and its value is 1 if and only if all conditions hold. The first type of feature function is for PWM. It is defined as below,

$$f\left(y_{j},y_{j-1},j,x\right)=x_{1,j}I\{y_{j}=u\},$$

where *u *is 0 or 1 which will be the label of that bin. It is the only type of real value function in CTF. The second kind of feature functions are designed to capture the occurrence of features. It is defined as

$$f\left(y_{j},y_{j-1},j,x\right)=I\{y_{j-1}=u\;and\;y_{j}=v\;and\;x_{i,j}=1\},$$

where both *u *and *v *are labels. The third type of feature function targets the co-occurrence of two histone markers and its definition is

$$f\left(y_{j},y_{j-1},j,x\right)=I\{y_{j-1}=u\;and\;y_{j}=v\;and\;x_{i,j}=1\;and\;x_{i^{\prime },j}=1\},$$

where *i *and *i' *corresponds to two histone markers. This kind of feature functions are able to capture co-occurring features such as bivalent domains[22] or "not in a promoter region or H3K4me3", which is a marker of active enhancer[23]. At last, feature functions to capture patterns in adjacent bins as a complement for above feature functions are defined as

$$f\left(y_{j},y_{j-1},j,x\right)=I\{y_{j-1}=u\;and\;y_{j}=v\;and\;x_{i^{\prime },j-1}=1\;and\;x_{i,j}=1\},$$

and

$$f\left(y_{j},y_{j-1},j,x\right)=I\left\{y_{j-1}=u\;and\;y_{j}=v\;and\;x_{i^{\prime },j-1}=1\;and\;x_{i,j}=1\;and\;x_{i^{\prime \prime },j+1}=1\right\}$$

where *i *and *i' *corresponds to two features and *u *and *v *are tags.

### Training

To estimate the parameter vector **λ**, we use a Regularized Maximum Conditional Log Likelihood method as

(1) $$\lambda_{ML}=\underset{\lambda }{\text{arg}\text{max}}\left(\text{ln}(p\left(y|x;\lambda \right)\right)$$

That is

(2) $$\lambda_{ML}=\underset{\lambda }{\text{arg}\text{max}}\left(\overset{L}{\underset{t=1}{\sum }}\lambda_{k}f_{k}-\text{ln}\left(Z\left(x\right)\right)-\frac{{∥\lambda ∥}^{2}}{2\sigma^{2}}\right)$$

where $Z\left(x\right)=\underset{y^{\prime }}{\sum }\text{exp}\left(\overset{L}{\underset{t=1}{\sum }}\overset{K}{\underset{k=1}{\sum }}\lambda_{k}f_{k}\left(y_{t}^{\prime },y_{t-1}^{\prime },x\right)\right)$ is the partition function and || || is the L-2 norm. In CTF, liblbfgs (http://www.chokkan.org/software/liblbfgs/), an open source library for unconstrained minimization, was used to find the optimal weight vector, **λ**.

### Prediction

To predict a label for each bin, we estimated the marginal probability of *j*-th bin to be TFBS as

$$s_{j}=p\left(y_{j}=1|x;\lambda \right),$$

which is assigned as the score of each bin. Thus, we can set a threshold and bins will be assigned as TFBSs if their scores exceed the threshold. The rest bins are assigned as background.

### Data

The binding sites of 13 transcription factors (TFs) in the mouse ES cells were obtained directly from the ChIP-seq data of Chen et al. [24] The 13 TFs were c-Myc, CTCF, E2f1, ESrrb, Klf4, Nanog, n-Myc, Oct4, Smad1, Sox2, STAT3, Tcfcp2l1 and Zfx. The position weight matrices (PWM) of TFs were obtained from JASPAR[28] and PWMs not available in JASPAR were obtained from Chen et al[24]. The locations of transcription start sites (Refseq mm8, April 8, 2012) were obtained from UCSC genome browser[25]. Also, the sequence of mouse genome (mm8, April 8, 2012) was downloaded from UCSC Genome Browser. Original ChIP-seq data on 8 distinct histone modification information was obtained from [26]. MACS[27] was employed with default parameters to call peaks from ChIP-seq data.

### Generating gold-standard TFBS dataset and feature matrix

"Peak-centric"[15] method was used to generate gold-standard dataset on the binned genome. First, mouse genome was divided into 200bp bins. Then, we assigned bins overlapped with the centers of TFBSs as positive ones and other bins as negative ones. Similar strategy was applied to generate a feature matrix (Additional file 1). The PWM score assigned to a certain bin was the maximal PWM score inside the bin. Then, for other features, the value corresponding to a histone modification of a certain bin was set to 1 if that bin overlapped with one peak and 0 otherwise. As for transcription start site (TSS) proximity, we defined the promoter region as a 4,000-bp interval centred at the TSS and if bins overlapped with that region, their values of TSS proximity were set to 1; otherwise, they were 0.

### Performance evaluation

In order to evaluate the performance of CTF, 10-fold cross-validation was employed. In the cross validation, 19 autosomes and chromosome X in mouse genome were randomly divided into 10 groups. Then, one group was utilized as test set and the rest as the training set. To measure the performance, we calculated Area Under the Curve (AUC) of Receiver Operator Characteristic (ROC) curve. ROC curve is a curve of True Positive Rate (TPR) vs. False Positive Rate (FPR) by changing the threshold of the model. For some methods, we were unable to get enough prediction to plot the complete ROC curve. Thus, in the comparison of all methods, we only computed the area under ROC curve when FPR was less than 10%, which was denoted as AUC_10%_. Another rationale was that in this range, the number of false positives was moderated and the model was useful.

We defined True Positives (TPs) as positive bins that were predicted as TFBSs and False Positives (FPs) as non-TFBS bins that were predicted as TFBSs. Similarly, negative bins predicted as non-TFBSs were termed True Negatives (TNs). Negative bins predicted as positives were defined as False Negatives (FNs). Then, True Positive Rate (TPR) was defined as the fraction of TPs called by a model in all positives. False Positive Rate (FPR) was defined as the fraction of FPs called by a model in all negatives.

In order to evaluate other methods with the same criterion, we put TFBSs predicted by other methods into bins according to their positions and the scores of those bins became the scores of corresponding TFBSs. If there were several TFBSs in one bin, the maximal score was chosen as the score of the bin. In this manner, we could measure the performance of all methods with the same criterion.

### Running other methods

We compared CTF with two existing methods and the baseline PWM method. The two existing methods were Chromia[13] and Cluster-Buster[21]. Chromia was downloaded from its website (http://tabit.ucsd.edu/download/Chromia2.tar.gz). Since the current release of Chromia contained the prediction result files generated from the same data set used in this paper, the results of Chromia was used directly. After this, predicted TFBSs were merged to bins and the results were then evaluated. Cluster-buster focused on detecting clustered motifs within a relatively narrow range, and did not consider epigenetic modification information. Cluster-Buster was run with parameters, "-c 1 -m 1 -g 20 -f 2". Position weight matrix (PWM) baseline method used solely the PWM score of every bin to identify TFBSs and we used various cut-offs to draw the ROC curves.

## List of abbreviations

TFBS (transcription factor binding site); ChIP-seq (chromatin immunoprecipitation followed by massively parallel sequencing); CRF (conditional random field); CTF (CRF-based TFBS finding system); TP (true positive); TN (true negative); FP (false positive); FN (false negative);. FPR (false positive rate); TPR (true positive rate); PWM (position weight matrix); ROC (receiver operating characteristic); AUC (area under the curve).

## Competing interests

The authors declare that they have no competing interests.

## Authors' contributions

CCW conceived and directed the whole project. YPH, GYZ, and CCW designed the framework of CTF system. YPH and CCW implemented the system. YPH and GYZ produced the test datasets. YPH and YZZ ran the tests and comparisons. YPH drafted the manuscript and all authors revised the manuscript. All authors read and approved the final manuscript.

## Supplementary Material

Additional File 1 **Formulation of TFBSs prediction problem**. TFBSs prediction problem can be formulated as a function to map a feature matrix (the above matrix in the figure) to an annotation (the below row vector). In the feature matrix, every row corresponds to one features and every column corresponds to one 200 bp bin in a genome. Feature types contain one real value feature (PWM) and multiple binary features (such as "is the bin within a promoter region" and "is it within the peak of a histone marker"). Note that "TSS" stands for transcription start site proximity. Click here for file

Additional File 2 **ROC curves for CTF, Chromia and PWM on the dataset of 13 transcription factors**. This figure, similar to Figure 5, contained the ROC curves of CTF, Chromia and PWM on all 13 transcription factors. CTF was the CTF model with all features and its ROC curve was obtained by using a 10-fold cross-validation procedure and changing the threshold. ROC curve of Chromia was calculated by using the same data and model contained in its release. ROC curve of PWM was got by scoring directly. Click here for file

Additional File 3 **Supplement tables**. Click here for file
