# Supplementary figures and images for: CTF: a CRF-based transcription factor binding sites finding system
Source: BMC Genomics. 2012 Dec 17;13(Suppl 8):S18. doi: 10.1186/1471-2164-13-S8-S18 (PMC3535700; doi:10.1186/1471-2164-13-S8-S18)

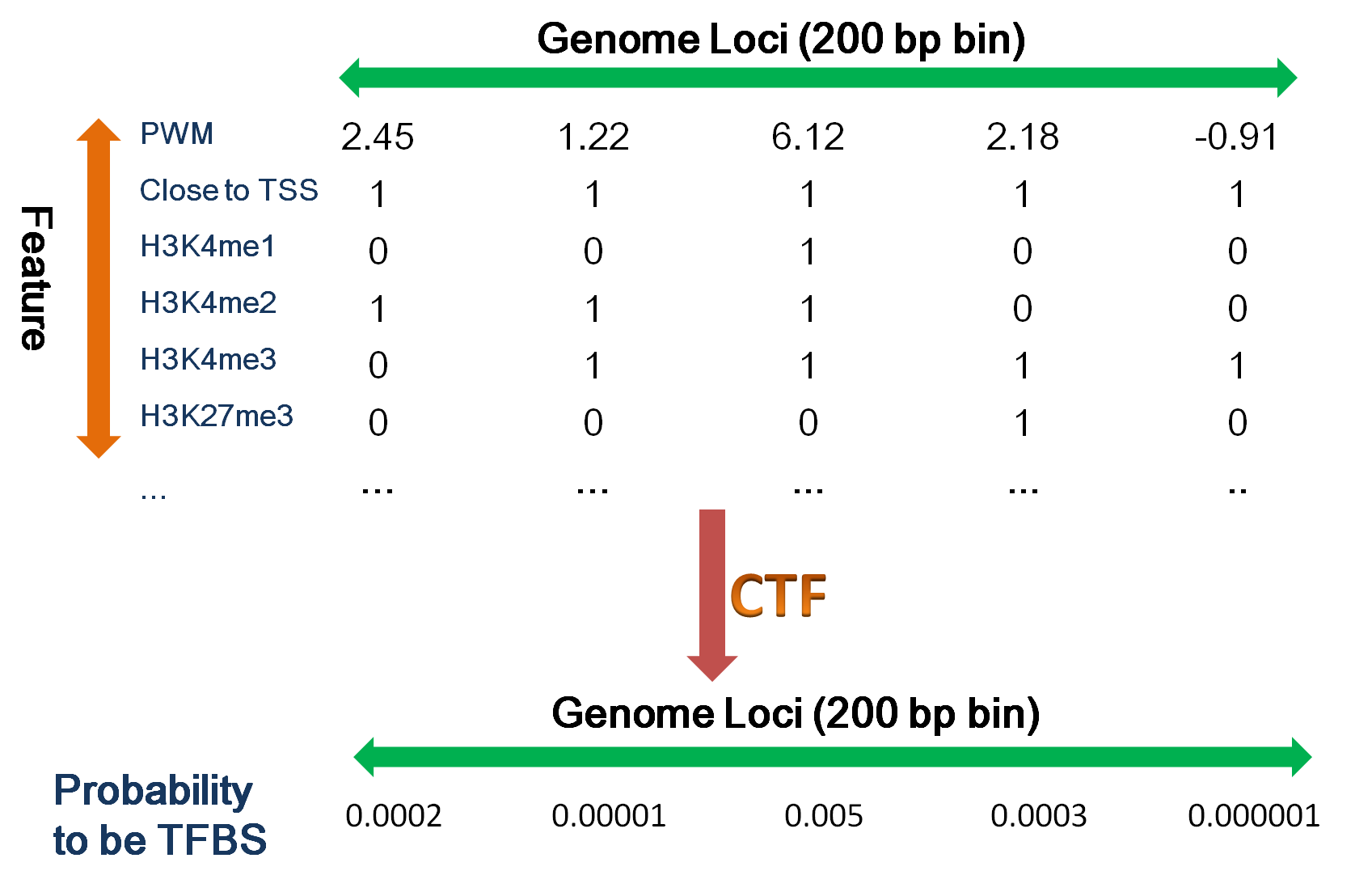

Supplement: Additional File 1 — Formulation of TFBSs prediction problem. TFBSs prediction problem can be formulated as a function to map a feature matrix (the above matrix in the figure) to an annotation (the below row vector). In the feature matrix, every row corresponds to one features and every column corresponds to one 200 bp bin in a genome. Feature types contain one real value feature (PWM) and multiple binary features (such as "is the bin within a promoter region" and "is it within the peak of a histone marker"). Note that "TSS" stands for transcription start site proximity. [file 1471-2164-13-S8-S18-S1.PNG]

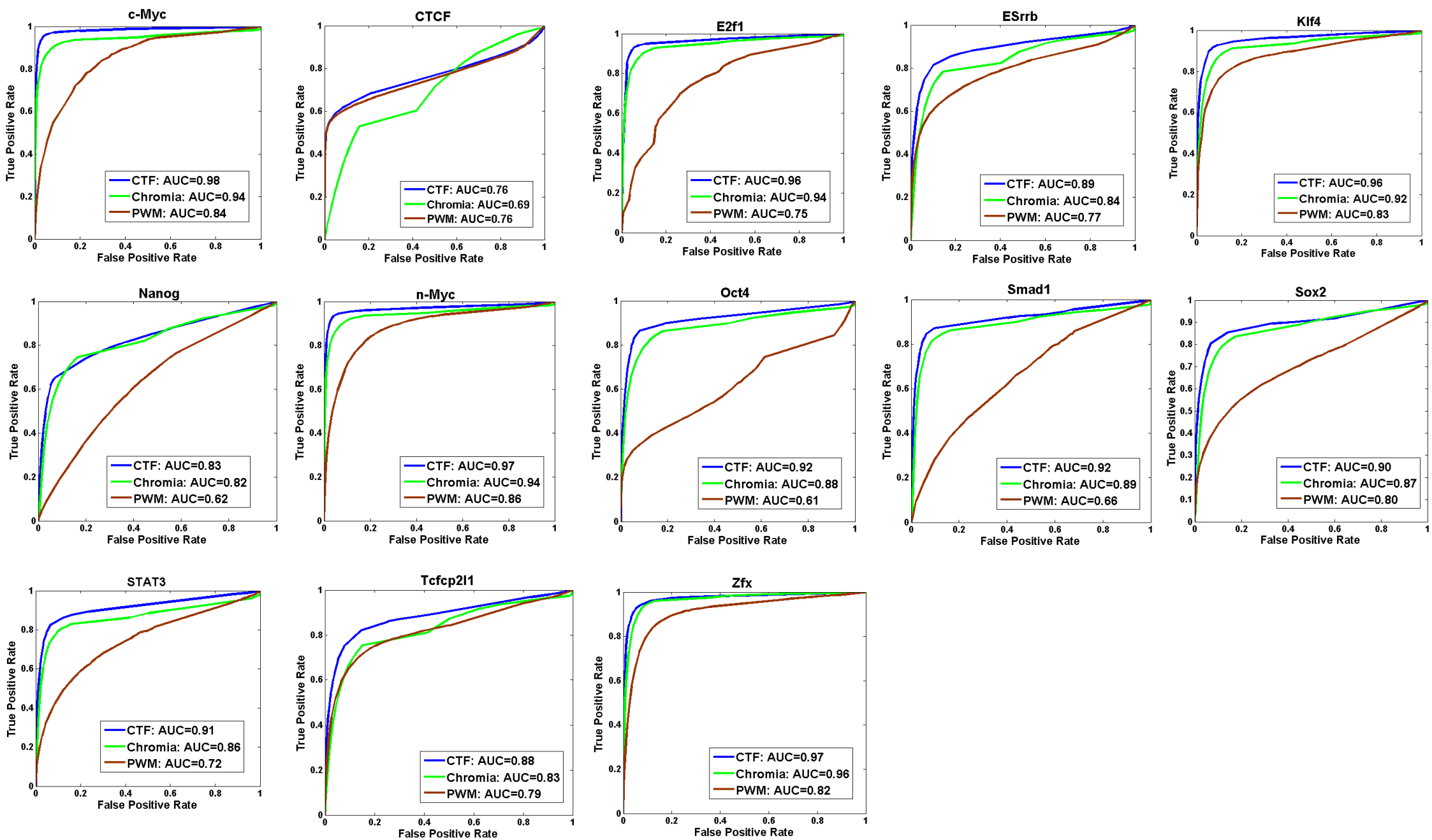

Supplement: Additional File 2 — ROC curves for CTF, Chromia and PWM on the dataset of 13 transcription factors. This figure, similar to Figure 5, contained the ROC curves of CTF, Chromia and PWM on all 13 transcription factors. CTF was the CTF model with all features and its ROC curve was obtained by using a 10-fold cross-validation procedure and changing the threshold. ROC curve of Chromia was calculated by using the same data and model contained in its release. ROC curve of PWM was got by scoring directly. [file 1471-2164-13-S8-S18-S2.PDF]
