# Supplementary material for: CTF: a CRF-based transcription factor binding sites finding system
Source: BMC Genomics. 2012 Dec 17;13(Suppl 8):S18. doi: 10.1186/1471-2164-13-S8-S18 (PMC3535700; doi:10.1186/1471-2164-13-S8-S18)
Supplement: Additional File 3 — Supplement tables. [file 1471-2164-13-S8-S18-S3.PDF]

Table S1 - Data sources of histone markers

Histone Markers	GEO id	Reference
H3K27me3	GSE12241	Mikkelsen et al[28]
H3K36me3	GSE12241	Mikkelsen et al [28]
H3K4me1	GSE11172	Meissner et al [26]
H3K4me2	GSE11172	Meissner et al [26]
H3K4me3	GSE12241	Mikkelsen et al [28]
H3K9me3	GSE12241	Mikkelsen et al [28]
H3	GSE12241	Mikkelsen et al [28]
H4K20me3	GSE12241	Mikkelsen et al [28]

Table S2 - Data of binding sites of 13 transcription factors

Transcription factors	# of peaks	# (percent) of peaks in promoter regions
c-Myc	3422	2397 (70%)
CTCF	39609	3960 (10%)
E2f1	20699	9922 (48%)
ESrrb	21647	3972 (18%)
Klf4	10875	4334 (40%)
Nanog	10343	945 (9%)
n-Myc	7182	4646 (64.69%)
Oct4	3761	964 (26%)
Smad1	1126	102 (9%)
Sox2	4526	499 (11%)
STAT3	2546	556 (21%)
Tcfcp2l1	26910	5272 (20%)
Zfx	10338	5648 (55)

Table S3 - The True Positive Rate of CTF, Chromia and PWM at 1% False Positive Rate on the data set of 13 transcription factors in mouse ES cell

Transcription factors	CTF	PWM	Chromia
c-Myc	**0.84**	0.19	0.67
CTCF	**0.52**	0.51	0.05
E2f1	**0.65**	0.11	0.52
ESrrb	**0.43**	0.31	0.16
Klf4	**0.65**	0.37	0.41
Nanog	**0.27**	0.04	0.11
n-Myc	**0.82**	0.29	0.67
Oct4	**0.45**	0.22	0.31
Smad1	**0.51**	0.05	0.19
Sox2	**0.45**	0.19	0.17
STAT3	**0.48**	0.15	0.28
Tcfcp2l1	**0.40**	0.27	0.17
Zfx	**0.73**	0.30	0.55
Average	**0.55**	0.23	0.33
